# Fragmenta de Viribus Medicamentorum positivis, sive in sano Corpore humano observatis

**Published:** 1834-10-01

**Authors:** 


*Fragmenta de Viribus Medicamentorum positivis, sive in sano Corpore humano observatis.* A Samuele Hahnemann, m.d. Edidit F. F. Quin, m.d., Medicus Ordinarius Leopoldi, Primi Regis Belgarum, &c.—London, 1834. 8vo. pp. 214.

Sir Gilbert Blane observes, in his Select Dissertations, (vol. i. p. 201 *et seq.* 2d edition,) that it would be an instructive thing to compare the results in a given disease, when nothing is done, with those which are obtained from the exhibition of remedies. In the present state of the medical art, however, he thinks it unjustifiable to leave the patient to the resources of nature; and therefore the comparison can be instituted only by observing what results were obtained in the infancy of our art, when inert remedies alone were often administered. He accordingly takes forty-two cases of acute disease, detailed by Hippocrates in two sections of one of his works, and finding that, of these, twenty-five terminated fatally, exults in the improvement of the medical art that has since taken place. Now Sir Gilbert appears to us to have misunderstood the scope of the work of the Father of Physic. It was not intended to give the average results of his method of treatment, but the more interesting cases which had occurred to him. The sage of Cos was not writing to get into practice, and therefore did not think it necessary to trumpet forth his successful cases; he selected those which were the most instructive, those in which the symptoms were the most severe, and the character of the disease consequently best developed. Hence these cases can by no means be quoted as showing the average result of medical practice B.C. 400.*

But what Hippocrates did not show, Hahnemann does. His method of giving a disguised nothing, a well-dressed and innocent zero to the patient, allows full scope to the *vis medicatrix naturæ*. It is on this account that we think homœopathy worth the attention of the most enlightened physician; for, if the followers of Hahnemann outrage common sense by their theory, they often appeal to it by their practice; and the results of the swallowed infinitesimals might have convinced Sir Gilbert Blane that if diseases, even acute ones, are let alone, the mortality will not be 25 in 42. It would be absurd, indeed, when the case clearly admits of being benefited by medicine, to follow the system of the nothingarians; but it may afford encouragement to the hypochondriac physician, who often fears both the disease and the remedy, to know that his maxim of *saltem non nocere* is a just one, and that it is not necessary to kill patients, on the supposition that the only alternative is to let them die. And thus it is that, as chemistry borrowed much that was valuable from alchemy, so homœopathy will throw new light on the practice of physic.

* It is pleasant to see the way in which a celebrated compiler has touched upon this subject. One might almost suppose that he imagined the forty-two cases to have formed the sum total of Hippocrates' practice. "Skilful and diligent in his profession, he openly declared the measures which he had taken to cure a disease, and candidly confesses that, of forty-two patients which were entrusted to his care, only seventeen had recovered, and the rest had fallen a prey to the distemper, in spite of his medical applications."—*Lempriere's Classical Dict.*, art. Hippocrates. 8vo. edit. 1809.

The work before us consists of Hahnemann's account of the effects of a number of remedies on the healthy body: these effects are apparently those produced by small doses; and he subjoins the symptoms as given by other authors, being apparently those resulting from large ones: thus, for example, Hahnemann gives the following account of the symptoms produced by cantharides.

"Lytta Vesicatoria L.

(*Pulveris Tinctura.*)

Vis.*

"Micturitio.
"Lotium guttatim destillans.
"Dolor pressorio lancinans in vesicæ collo.
"Formicatio et pruritus in urethrâ inter mictum.
5 "Dolor mordax in urethrâ inter mictum.
"Dolor strictorius in artibus, fere paralyticus.
"Dolor in affectâ parte (v. c. ulcere) lacerans.
"Dolor lacerans in dorso.
"Effluvium auctum ex affectâ parte (v. c. ex ulcere pedum, e naribus in coryzâ chronicâ, ex urethrâ in blennorrhœa chronicâ.)
10 "Muci in coryzâ chronicâ in sanguineum mutatio.
"Sensus mordax in oculis, quasi a sale culinari insperso.
"Inappetentia ciborum.
"Debilitas, prostratio virium.
"Morositas.
15 "Diarrhœa sine torminibus.
"Tormina.
"Incarceratio flatuum in hypochondriis.
"Agrypnia.
"Pruritus in cute.
20 "Sudor lenis nocturnus." (P. 47.)

After this, Brassavola, Lange, Paschalius, Occo, Pareus, Vierus, Joach. Camerarius, and others, testify that cantharides will cause bloody urine, priapism, inflammation of the penis, &c. &c.

This work is certainly of some use, as well as a medical curiosity; and it may be consulted with advantage as an index of cases of poisoning occurring in the older writers.

* "Vires hujus remedii durant ultra dies quatuordecim.—*Ed.*"

